# The Janus Kinase (JAK) FERM and SH2 Domains: Bringing Specificity to JAK–Receptor Interactions

**DOI:** 10.3389/fendo.2017.00071

**Published:** 2017-04-18

**Authors:** Ryan Ferrao, Patrick J. Lupardus

**Affiliations:** ^1^Department of Structural Biology, Genentech Inc., South San Francisco, CA, USA

**Keywords:** JAK1, JAK2, JAK3, TYK2, Interferon, FERM, SH2, Janus kinase

## Abstract

The Janus kinases (JAKs) are non-receptor tyrosine kinases essential for signaling in response to cytokines and interferons and thereby control many essential functions in growth, development, and immune regulation. JAKs are unique among tyrosine kinases for their constitutive yet non-covalent association with class I and II cytokine receptors, which upon cytokine binding bring together two JAKs to create an active signaling complex. JAK association with cytokine receptors is facilitated by N-terminal FERM and SH2 domains, both of which are classical mediators of peptide interactions. Together, the JAK FERM and SH2 domains mediate a bipartite interaction with two distinct receptor peptide motifs, the proline-rich “Box1” and hydrophobic “Box2,” which are present in the intracellular domain of cytokine receptors. While the general sidechain chemistry of Box1 and Box2 peptides is conserved between receptors, they share very weak primary sequence homology, making it impossible to posit why certain JAKs preferentially interact with and signal through specific subsets of cytokine receptors. Here, we review the structure and function of the JAK FERM and SH2 domains in light of several recent studies that reveal their atomic structure and elucidate interaction mechanisms with both the Box1 and Box2 receptor motifs. These crystal structures demonstrate how evolution has repurposed the JAK FERM and SH2 domains into a receptor-binding module that facilitates interactions with multiple receptors possessing diverse primary sequences.

## Introduction

Cytokines are a large family of secreted proteins with wide-ranging effects on cell growth, hematopoiesis, immunity, and inflammation (1, 2). Cytokines induce signaling by binding a specific set of cognate transmembrane receptors, a process that is thought to be initiated either by *de novo* dimerization of these receptors or by a conformational rearrangement of existing inactive receptor dimers (3, 4). Assembly of a functional cytokine–receptor signaling complex results in the activation and *trans*-phosphorylation of Janus kinases (JAKs), a family of cytoplasmic multi-domain tyrosine kinases (5). The four members of the JAK family, JAK1, JAK2, JAK3, and TYK2, are constitutively associated with the intracellular domains of cytokine receptors. Activated JAKs phosphorylate tyrosine residues within the cytokine receptor intracellular domains, which serve as binding sites for the STAT (signal transducer and activator of transcription) transcription factors (6). Recruitment of STATs to the receptor–JAK complex facilitates STAT phosphorylation by active JAKs, leading to STAT dimerization and translocation to the nucleus, where they promote transcription of cytokine-responsive genes.

Cytokine receptors can be divided into the class I and class II subfamilies based on structural similarities in both their ligands and their receptor sequences (7). The class I cytokine receptor family, which contains a conserved extracellular WSXWS motif within the second FNIII domain of the cytokine-binding homology region (8, 9), is made up of four subfamilies. Three of the subfamilies, the common gamma (γ_c_) family, the common beta (β_c_) family, and the gp130 family, are defined by their use of a shared (or common) signal transducing chain for the formation of homo- and hetero-oligomeric signaling complexes (10). An additional subfamily of class I cytokine receptors, which includes the growth hormone (GH) and erythropoietin (EPO) receptors, signal as homodimeric pairs (8, 11). The class II cytokine receptor family contains receptors for the type I, type II, and type III interferons as well as the IL-10 cytokine family (12). Each cytokine receptor family interacts with and signals through a distinct subset of JAKs. GH and EPO receptors signal solely through JAK2, the β_c_ family through JAK2, the γ_c_ family through JAK1 and JAK3, and the gp130 and class II cytokine families through JAK1, JAK2, and/or TYK2 (7).

The intracellular domains of cytokine receptors are largely unstructured (13) and contain anywhere from several dozen to several hundred amino acids. Signaling through cytokine receptors was initially found to require the membrane proximal region of the intracellular domain (14), which was subsequently further defined as two distinct conserved motifs: a proline-rich segment termed “Box1” and a hydrophobic segment approximately 20–40 residues downstream called “Box2” (15, 16). The JAK kinases were identified in the early 1990s (17–20) and were quickly found to be essential for mediating signaling downstream of cytokine receptors (21–23). In addition to their C-terminal kinase domain, JAKs also contain a pseudokinase domain (psKD), an SH2 domain, and a FERM domain. Individual JAK family members were found to physically associate with various cytokine receptors (24–27), and the JAK FERM and SH2 domains were identified as essential for mediating the JAK–receptor interaction (28–34). More recently, the structure of the pseudokinase and kinase domains has been solved, elucidating the structural basis for regulation of kinase activity by the pKD (35–38).

Due to the essential role of JAK–receptor interactions in cytokine signaling, naturally occurring mutations in both cytokine receptors and JAKs lead to immunodeficiency and myeloproliferative disorders (39). An autosomal severe combined immunodeficiency (SCID)-associated mutation, Y100C, is located in the hydrophobic core of the JAK3 FERM domain. This mutation disrupts the interaction with the γ_c_ receptor chain and leads to constitutive JAK3 phosphorylation, likely due to aggregation-induced *trans*-phosphorylation of the destabilized JAK3 (40). Additionally, nonsense mutations that result in a truncated γ_c_ lead to X-linked SCID, and the L271Q mutation in the Box1 of γ_c_ causes a more moderate form of X-linked combined immunodeficiency (41, 42). Despite a substantial amount of research performed on understanding the essential components of both the receptor and kinase, the molecular underpinnings of the JAK–receptor interaction have remained elusive. This review will focus on the recent advances in our understanding the molecular structure of JAK–receptor complexes.

## Structure of the JAK Family FERM–SH2 Receptor Interaction Module

**Figure 1:**
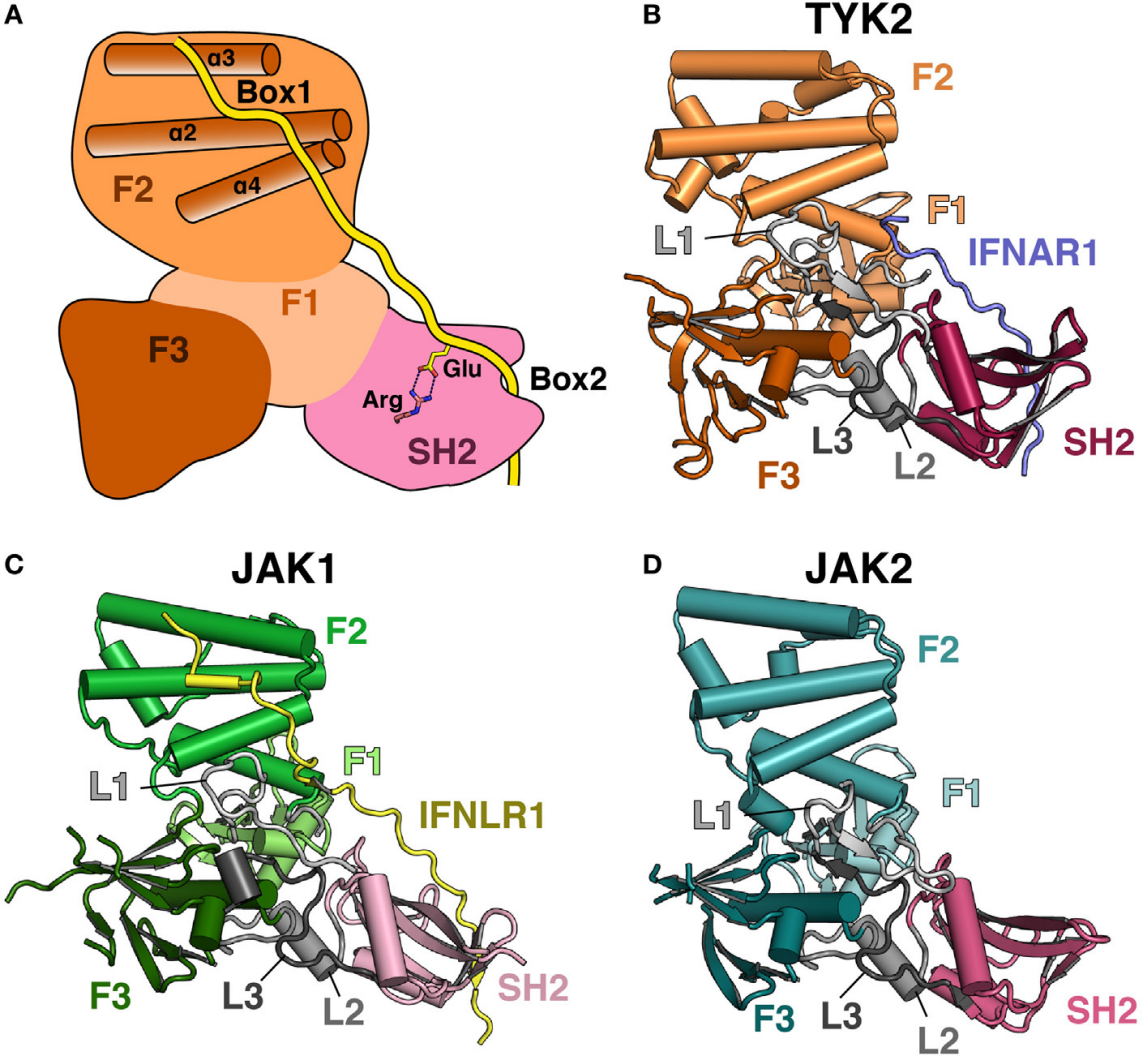
**Comparison of Janus kinase (JAK) FERM/SH2 domains**. **(A)** Cartoon depiction of a JAK FERM and SH2 module bound to a receptor, illustrating receptor recognition principles discovered in recent crystal structures, followed by representations of crystal structures solved for **(B)** TYK2 (PDB ID 4PO6), **(C)** JAK1 (PDB ID 5L04), and **(D)** JAK2 (PDB ID 4Z32). The F1, F2, and F3 FERM subdomains are colored in various shades of orange, green, and blue, respectively. SH2 domains are colored in various shades of pink. Linkers L1, L2, and L3 are colored in various shades of gray. The IFNAR1 receptor peptide is colored blue, and the IFNLR1 is in yellow.

An atomic-level structural model of the JAK FERM and SH2 domains and the mechanism of JAK interaction with receptors remained elusive until recently, largely due to the intractability of JAK protein crystallization due to poor expression and solubility (43). Copurification with a stabilizing receptor enabled an initial X-ray crystal structure of the human TYK2 FERM and SH2 domains in 2014 (44) followed by crystal structures of the human JAK1 (45, 46) and JAK2 (47) FERM–SH2 fragments in 2016. These structures surprisingly revealed that the JAK FERM and SH2 domains are tightly associated to form a single receptor-binding module (Figures 1A–D). The overall structural organization of the JAK FERM is similar to canonical FERM domains and consists of three subdomains, the ubiquitin-like F1, acyl-CoA-binding protein-like F2, and pleckstrin homology domain-like F3, using the FERM domain terminology established for focal adhesion kinase (FAK) (48). These subdomains pack into a canonical trilobed FERM architecture (Figure 1A). While the overall domain topology is conserved when compared to FERM domains from the ezrin/radixin/moesin and FAK families, JAK FERMs posses several unique differences that enable an intimate interaction with the SH2 domain. First, an elongated linker, L1, connects the F1 and F2 domains and forms a large part of the interaction surface between the FERM and SH2 domains. In classical FERMs, L1 is typically between 13 and 15 residues but has extended to between 29 and 42 residues in JAKs. In addition to the L1, the SH2 domain packs against the F1-α1 helix, the highly conserved F3–SH2 linker (L2) and the SH2–pKD (L3) linker (44).

**Figure 2:**
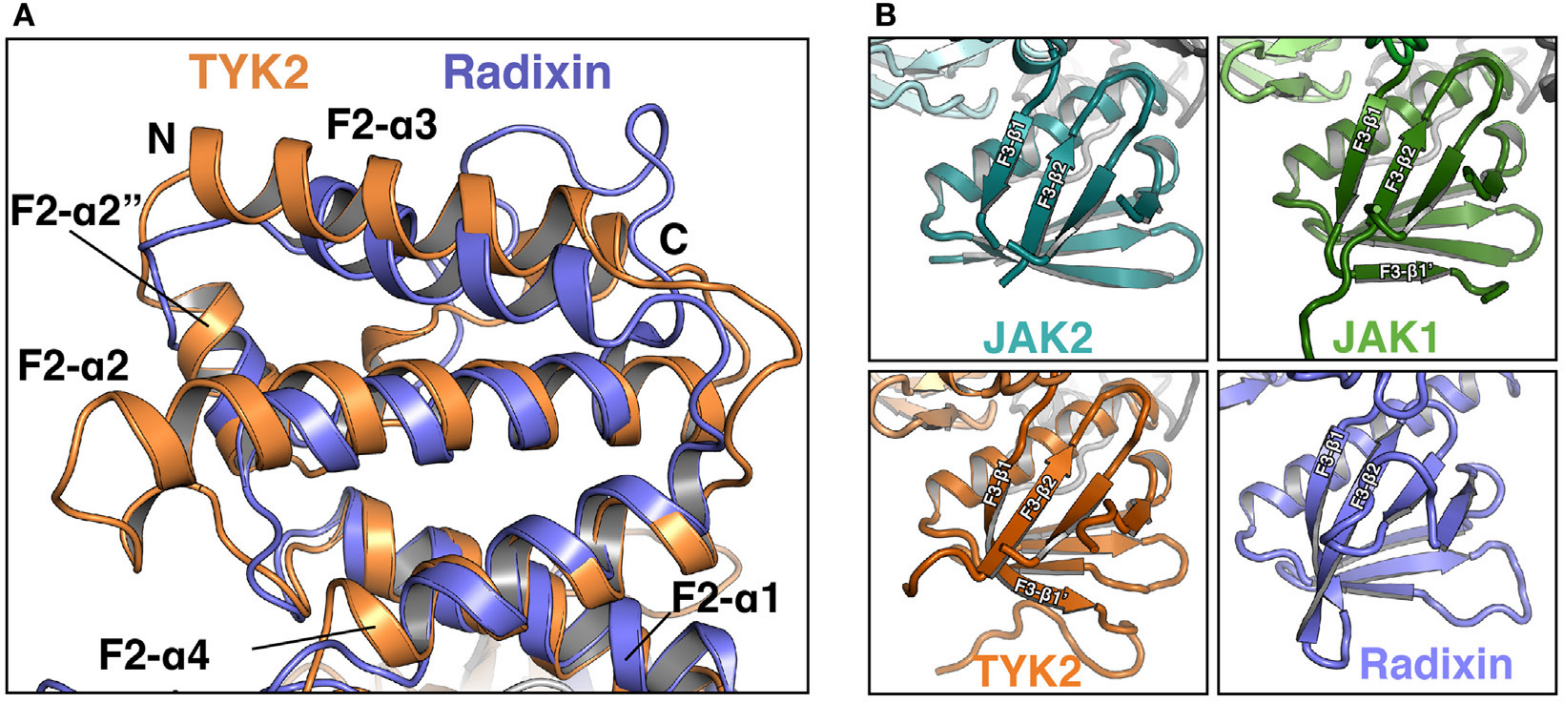
**Comparison of Janus kinase (JAK) and ERM family FERM domains**. **(A)** Comparison of the FERM F2 subdomains of TYK2 (orange) and radixin (blue, PDB ID 1GC7). TYK2 and radixin F2 domains were superposed in PyMol with a root-mean-square deviation of 2.98 Å over 545 atoms. **(B)** Comparison of the F3 subdomains of JAK2 (top left, teal), JAK1 (top right, green), TYK2 (bottom left, orange), and radixin (bottom right, blue, PDB ID 1GC7).

The F2 subdomain of the JAK FERM also deviates significantly from classical FERM domain structure. Additional residues in the linker between F2-α1 and F2-α2 allow for the extension of the N-terminal end of F2-α2 helix by one turn in JAK2 and two turns in TYK2 and JAK1 (Figure 2A). The C-terminal end of the F2-α2 and the N-terminal end of F2-α3 helices are also extended by one turn in JAK FERMs. The elongation of these two helices is likely related to the ability of the JAK FERM domains to utilize this interface to bind to cytokine receptors (discussed below). Additionally, the F2-α3 helix of JAKs is highly basic (44, 45), while in canonical FERM domains this chemical character is not preserved (49). The basic residues in this helix create a large positively charged patch on the JAKs, which may enable favorable interactions with the plasma membrane.

The core structure of the JAK F3 subdomain is highly similar to that of canonical FERM domains, consisting of a four-stranded β-sheet (β1–β4) sandwiched against a three-stranded β-sheet (β5–β7) followed by an α-helix (F3-α1) (Figure 2B). Yet interestingly, several loops within the F3 subdomain vary significantly between the JAK isoforms [for a comprehensive alignment of JAK family members, see Ref. (44)]. The first loop, between F3-β1 and F3-β2 strands, contains 12 amino acids in JAK2 and is largely disordered in this crystal structure (Figure 2B, top left). In JAK1, the F3-β1/β2 loop contains 22 amino acids and forms a β hairpin that packs against F3-β7, extending the β-sheet by a single strand, F3-β1′ (Figure 2B, top right). In TYK2, the loop is 35 amino acids and also forms a single strand that packs against F3-β7, with an additional loop visible (Figure 2B, bottom left). The C-terminal half of the linker in TYK2 is unstructured. The large insertions at this position are specific to JAK family FERM domains, as classical FERM domains contain only a short loop at this position (Figure 2B, bottom right). An additional disordered loop of variable length is located in between F3-β3 and F3-β4. F3-β3/β4 linker lengths are 12, 34, and 44 amino acids in JAK2, JAK1, and TYK2, respectively. These insertions are also not present in canonical FERM domains. Interestingly, both the length and sequence identities of these JAK F3 insertions are highly conserved between all higher eukaryotic species.

The overall structure of the JAK family SH2 domain is reminiscent of a canonical phosphotyrosine (pTyr)-binding SH2 domain, consisting of a central β-sheet flanked by two α-helices. In canonical SH2 domains, two loops flanking the SH2-αB helix form a hydrophobic groove that is the binding site for specificity determining residues at positions +3 and +5 relative to the pTyr (50). In the JAK SH2 domain, the SH2-EF loop and a β-hairpin formed by SH2-βG1 and SH2-βG2 create a large hydrophobic slot that is key for receptor Box2 binding (discussed below). In addition, the conserved, phosphate-coordinating arginine residue located at the base of the pTyr binding pocket is conserved in all JAKs except TYK2, where it has been replaced with a histidine residue.

## The Structure of TYK2 Bound to IFNAR1 Box2

**Figure 3:**
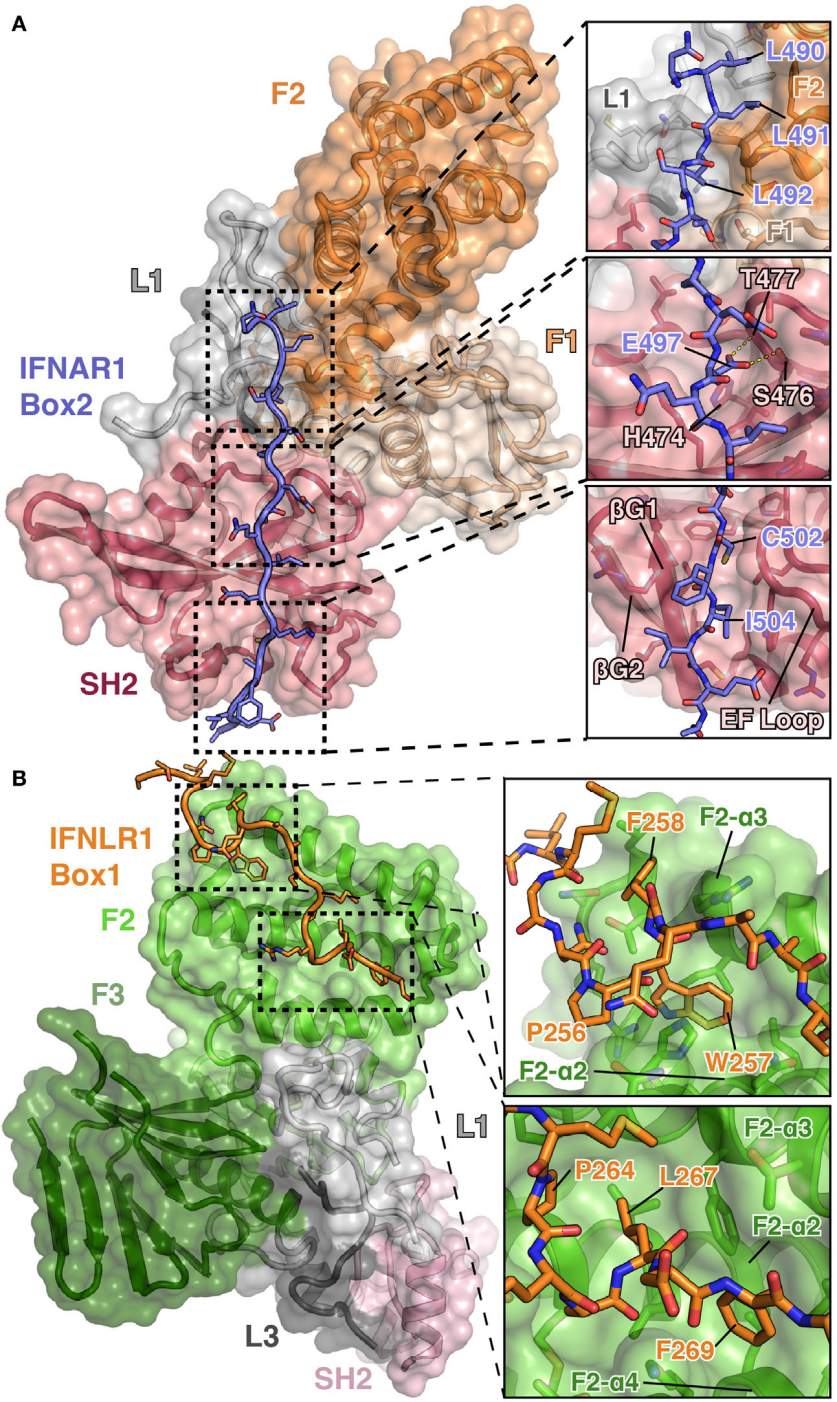
**Detailed views of the interactions between TYK2–IFNAR1 Box2 and JAK1 IFNLR1 Box1**. **(A)** Overview of the interaction between TYK2 FERM and SH2 (colored as in Figures 1A,B) domains in complex with the IFNAR1 Box2 region (left, PDB ID 4PO6). Detailed interactions are shown for three regions of the TYK2–IFNAR1 complex (right panels). In the N-terminal tri-leucine region, Leu490, Leu491, and Leu492 of IFNAR1 pack against a hydrophobic groove between the TYK2 F1 (tan), L1 (gray), and F2 (orange) domains (top panel). In the center of the interaction, Glu497 of IFNAR1 forms hydrogen bonds with Ser476 and Thr477 (middle panel). At the C-terminus of IFNAR1, Cys502 and Ile504 insert into a groove between the β-G1/2 hairpin and EF loop of the TYK2 SH2 domain (bottom panel). **(B)** Overview of the bipartite interaction between JAK1 FERM and SH2 (colored as in Figure 1C) domains in complex with the IFNLR1 Box1 region (left, 5IXD). Detailed interactions are shown for two regions of the JAK1–IFNLR1 complex (right panels). At the first site, IFNLR1 residue Trp257 inserts itself into a groove between JAK1 F2-α2 and F2-α3 (top panel). At the second site, hydrophobic residues Pro264, Leu267, and Phe269 of IFNLR1 pack into a groove formed by JAK1 F2-α2, F2-α3, and F2-α4 (bottom panel).

Structural studies of the JAKs bound to their receptors have historically been hampered by poor expression and solubility of the JAKs as well as weak affinities for their receptors. To overcome these biochemical hurdles, a construct containing a juxtamembrane peptide from IFNAR1 fused to the C-terminus of the TYK2 FERM and SH2 domains was used to generate the TYK2–IFNAR1 complex (44). Prior studies had shown that IFNAR1 is not trafficked to the cell surface in the absence of TYK2 (51), suggesting a strong constitutive interaction between these two molecules. This approach greatly enhanced the expression and solubility of the TYK2 FERM–SH2 and enabled crystallization of TYK2 with a peptide encompassing the Box2 sequence (residues 478–512) of IFNAR1 (44). Importantly, this structure revealed the Box2 of IFNAR1 bound in an extended conformation to a composite interface on the surface of the TYK2 FERM–SH2, with the bulk of the peptide interacting with the SH2 domain (Figure 3A).

The overall topology of the IFNAR1 Box2 interaction with TYK2 resembles that of a pTyr-containing peptide bound to an SH2 domain and can be broken down into three sections, all of which are important for stable interaction with TYK2 (44). At the N-terminus of the Box2 motif, triplet leucine residues, Leu490, Leu491, and Leu492, participate in a key interaction by packing against a hydrophobic groove formed between F2, L1, and F1 (Figure 3A, top panel). The next segment of IFNAR1 binds to what would be the pTyr binding pocket in a canonical SH2 domain (Figure 3A, middle panel). However, in place of a pTyr residue, a conserved glutamate (Glu497) is placed in the pocket. In TYK2, the arginine residue responsible for forming a salt bridge with the phosphate group in classical SH2 domain interactions is replaced with a histidine residue (His474), which does not participate in the interaction with IFNAR1. Instead, the sidechain carboxylate of Glu497 forms hydrogen bond interactions with residues Ser476 and Thr477 of TYK2, mimicking a subset of the contacts normally made by the phosphate group (Figure 3A, middle panel). TYK2 is unique among human JAKs, as all other family members maintain an arginine at this position, although mutagenesis of this Arg has been shown to not affect subcellular localization or function (52). In the third segment, the hydrophobic Box2 sequence of IFNAR1 forms a short β sheet with the βG1 strand of the TYK2 SH2 domain (Figure 3A, bottom panel). Two hydrophobic residues located at the +5 and +7 positions relative to Glu497, Cys502 and Ile504, pack into the hydrophobic groove formed between the SH2-EF loop and the SH2-βG1/2 hairpin. Importantly, this interaction is primarily mediated by backbone H-bond and aliphatic sidechain van der Waals interactions, therefore revealing how JAK SH2 domains can bind a range of Box2 sequences that contain aliphatic residues in the first and third positions.

## The JAK1 FERM Domain Interaction with Box1 Sequences from Class II Receptors

The structure of TYK2 bound to IFNAR1 only revealed the binding site for residues near the Box2 peptide; therefore, further structural characterization was required to elucidate the JAK binding site for the Box1 motif. To this end, crystal structures of the JAK1 FERM–SH2 bound to the Box1 of IFNLR1 and IL10R1 were determined (45), utilizing a co-expression and screening approach to identify tight binding receptors amenable to crystallization. Rather than expressing the FERM–SH2 and receptor as a fusion as with TYK2 and IFNAR1, the JAK1 FERM–SH2 and receptor were co-expressed as His and GST tag fusion proteins, respectively. This method identified IFNLR1 and IL10R1 as receptors that bound with high enough affinity to enable copurification of a JAK–receptor complex that could be utilized in crystallization trials (45).

**Figure 4:**
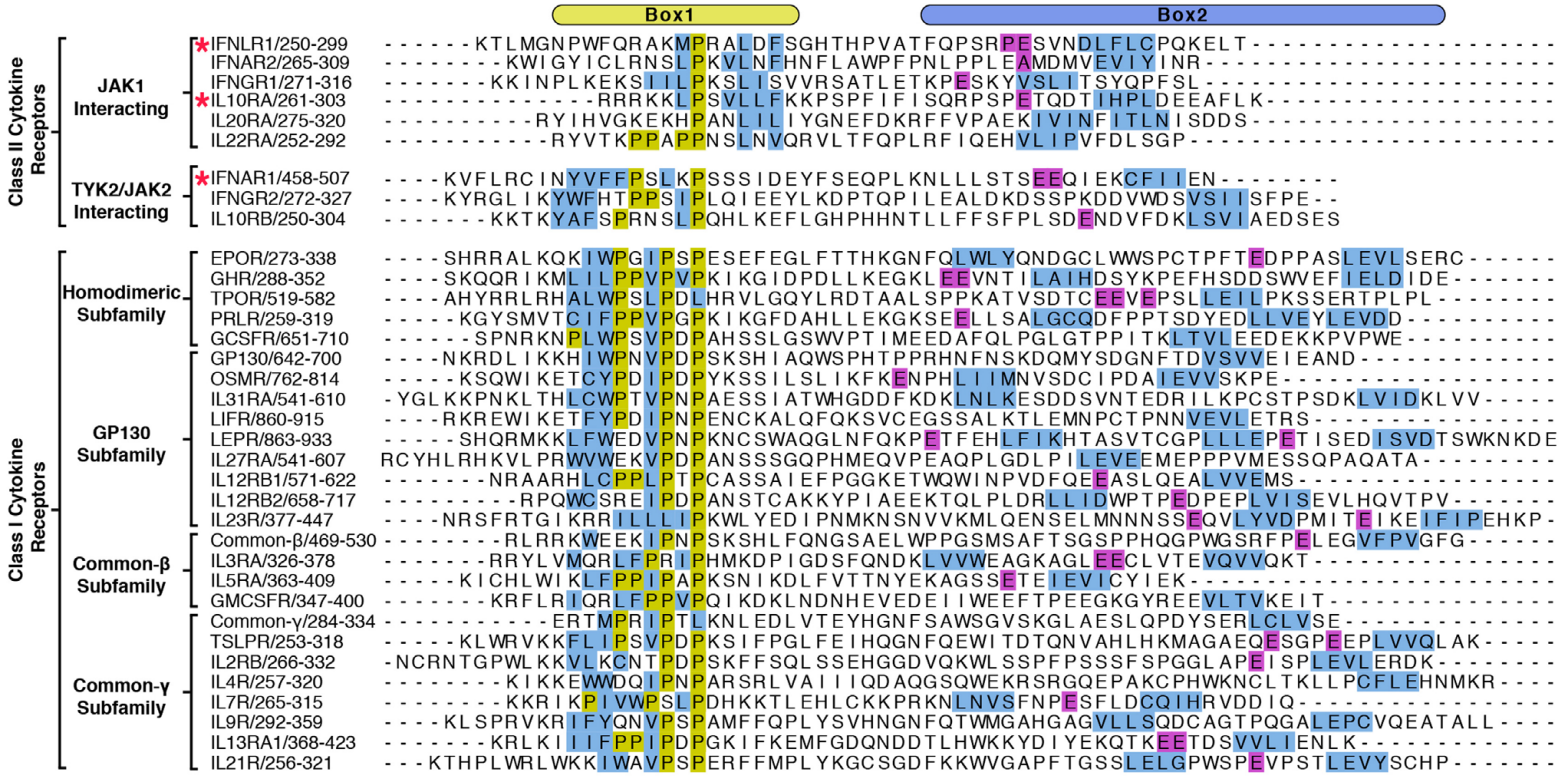
**Alignment of Box1 and Box2 motifs from Janus kinase (JAK)-interacting cytokine receptors**. The juxtamembrane intracellular sequences from all known signaling receptors that interact with JAKs were aligned by their Box1 proline motifs. Receptors are grouped by class I and class II, followed by subgroupings by either the JAK they interact with (class II) or by subfamily (class I). Due to the low sequence homology between receptors, the alignments were produced by hand, and the residues colored in order to highlight some of the shared features of the receptors. Hydrophobic residues within the Box1 region are colored in blue and prolines in yellow. For the Box2 region, putative four-residue hydrophobic motifs (aliphatic-X-aliphatic-X) are colored in blue, with glutamate residues three to seven residues N-terminal to these motifs are colored in purple. Receptors with known structures (IFNAR1, IFNLR1, and IL10RA) are marked with a star.

Importantly, these structures revealed a novel binding site for the Box1 motif of class II cytokine receptors. The Box1 of IFNLR1 binds to the F2 subdomain of the JAK1 FERM, in a cleft formed by helices F2-α2, F2-α3, and F2-α4 (Figure 3B). Interestingly, the location of the Box1 binding site was previously hypothesized, based on CONSURF-predicted sequence conservation within that region (53) and the effects of F2 destabilization on receptor binding (54). Like the IFNAR1 Box2 peptide interaction with TYK2, the IFNLR1 Box1 interface with JAK1 can be subdivided into multiple distinct interaction sites. The first is defined by Pro256, Trp257, and Phe258, which fold into a 3_10_ helix that facilitates the insertion of Trp257 into a deep groove formed by the F2-α2 and α3 helices (Figure 3B, top panel). Following Phe258, the IFNLR1 Box1 breaks contact with JAK1, rejoining at a second site defined by the motif PxxLxF (45). This motif folds into a second 3_10_ helix, aligning Pro264, Leu267, and Phe269 into a hydrophobic ridge that interacts with a groove formed by the F2-α2, α3, and α4 helices in the JAK1 FERM domain (Figure 3B, bottom panel). The proline and leucine residues found in this PxxLxF motif are completely conserved in all type II cytokine receptors, while the phenylalanine is a favored residue (Figure 4) (45). Indeed, in a second structure of a hybrid receptor formed by an N-terminal fusion of IFNLR1 “PWF” motif with the IL10R1 Box1 “PxxLxF” and Box2 motifs, the IL10R1 Box1 sequence (PSVLLF) bound in a nearly identical conformation to that seen for IFNLR1 (45).

Unlike TYK2, the JAK1 FERM–SH2 domain was soluble when purified in the absence of a receptor, enabling biophysical studies of receptor binding and granular dissection of the Box1 interaction. Quantitative biophysical measurement of the interaction between JAK1 and IFNLR1 peptide containing both Box1 and Box2 by Biolayer Interferometry established a binding affinity (*K*_D_) of 70 nM. The isolated IFNLR1 Box1 binds to JAK1 with an affinity of 1.2 µM, while the isolated Box2 does not measurably interact (45). These results indicate that while both Box1 and Box2 contribute to the JAK–IFNLR1 interaction, Box1 is the primary driver of the interaction. At this point, it is unknown if this observation will apply to interaction between other JAKs and receptors.

## Structure of JAK1 Bound to IFNLR1 Box1 and Box2

**Figure 5:**
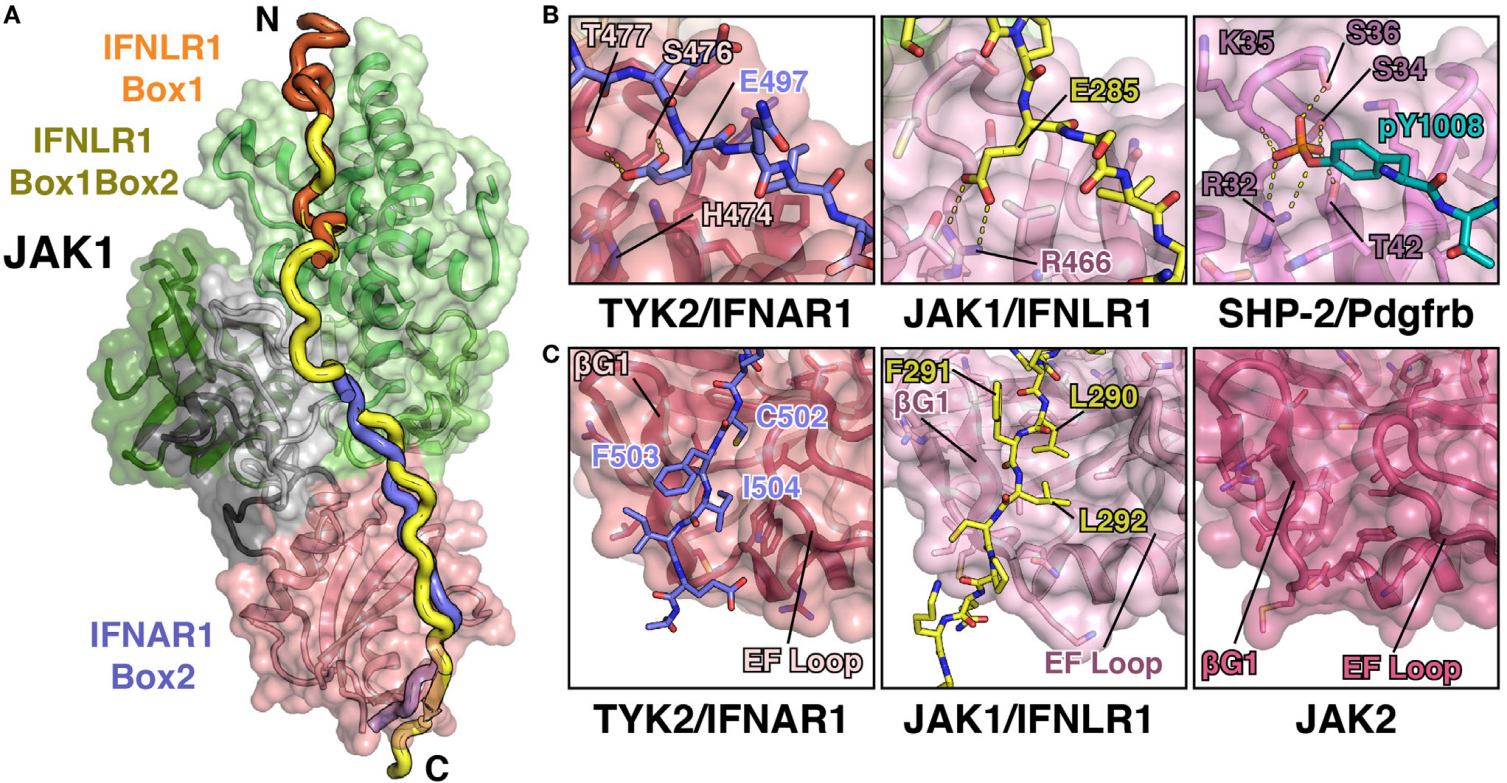
**JAK1 and TYK2 recognize the Box2 motif *via* a conserved mechanism**. **(A)** Cartoon representation of JAK1 (colored as in Figure 1C) bound to IFNLR1 receptor peptide (yellow) containing both the Box1 and Box2 motifs (PDB ID 5L04). For comparison, the IFNLR1 Box1 motif (orange) and the IFNAR1 Box2 (blue) are shown after superposition of JAK1 and TYK2, respectively. **(B)** Detailed comparison of the pTyr/Glu binding pocket of the TYK2 (PDB ID 4PO6), JAK1, and SHP-2 (PDB ID 1AYA) SH2 domains. TYK2 (left) contains a histidine residue (H474) at the base of the pocket, while JAK1 and SHP-2 have an arginine at this position that forms a salt bridge with either the glutamate in IFNLR1 or phosphate group in the PDGF receptor peptide. **(C)** Detailed comparison of the Box2 binding groove of TYK2, JAK1, and JAK2. IFNAR1 residues Cys502 and Ile504 pack into a groove in the TYK2 SH2 domain between the β-G1/2 hairpin and EF loop (left). In the JAK1/IFNLR1 structure, Leu290 and Leu292 pack into this groove (center). The SH2 domain of JAK2 (PDB ID 4Z32) maintains this conformation in the absence of receptor (right).

An important second crystal structure of the JAK1 FERM–SH2 bound to a peptide containing both the Box1 and Box2 domains of IFNLR1 has also been published (Figure 5A) (46). This structure, which was produced using the JAK1 FERM–SH2 linked to the IFNLR1 peptide at the C-terminus, for the first time demonstrates simultaneous binding of both Box1 and Box2 sites to a single JAK monomer. The model obtained from this structure confirms both the Box1 interaction site identified for JAK1 as well as authenticates key conserved features of the Box2 interaction identified in the TYK2–IFNAR1 structure (44). In addition, it corroborates the affinity measurements that indicate IFNLR1 Box1 and Box2 cooperate to create a high affinity 1:1 interaction with JAK1 (45).

As mentioned above, the structure of the IFNLR1 Box1 region is nearly identical to that of previously solved structures of JAK1–IFNLR1 and JAK1–IL10R1 Box1 peptides (45). Five additional linker residues that bridge the Box1 and Box2 regions are also present in the structure, although the electron density is relatively weak for this region due to a lack of peptide contacts with JAK1. More importantly, the key features of the JAK1–IFNLR1 Box2 region are conserved to the TYK2–IFNAR1 structure, yet with several notable differences (Figure 5B). First and foremost, like IFNAR1, IFNLR1 also contains a glutamate residue (Glu285) that extends into the SH2–pTyr binding pocket (Figure 5B). Yet unlike in TYK2, the canonical pTyr binding SH2 arginine residue (Arg466) in JAK1 forms a salt bridge interaction with Glu285. This closely approximates the interaction between the SH2 arginine and phosphate group in a canonical SH2–pTyr interaction, represented here by the SHP-2/Pdgfrb complex structure (Figure 5B). This interaction, while slightly different in topology to the TYK2–IFNAR1 Glu497 interaction, further supports the hypothesis that the JAK family SH2 domain evolved from an archaic precursor that participated in conditional receptor interactions regulated by phosphorylation (44).

Similarities between JAK1 and TYK2 peptide recognition extend to other sites identified in the TYK2–IFNAR1 interaction. The key “segment 1” leucine triplet found in IFNAR1 is changed to an Ala–Thr–Phe sequence in IFNLR1, yet it maintains a similar binding orientation. IFNLR1 Ala277 and Thr278 pack into the F2/L1 pocket in a similar conformation to IFNAR1 Leu490 and Leu491, while Leu492 of IFNAR1 is replaced by Phe279 in IFNLR1 and binds to the same pocket at the interface of the F1 and SH2 domains. While these binding triplets have a similar interaction phenotype, the gap between the IFNAR1 Leu492/IFNLR1 Phe279 residue and the SH2-interacting glutamate is six residues in IFNLR1 rather than the five residues seen in IFNAR1. This has profound effects on the backbone positioning of the peptide, which bulges out to accommodate the extra amino acid (46).

Additionally, the interaction between the hydrophobic Box2 and SH2 domain is conserved in the JAK1–IFNLR1 structure (Figure 5C). Like IFNAR1, the Box2 motif in IFNLR1 begins five residues C-terminal to the conserved glutamate (Glu285), with the +5 and +7 residues (Leu290 and Leu292) buried in a groove formed by the SH2-EF loop and βG1 strand. The Box2 motif also caps an antiparallel sheet formed with the βG1 and βG2 strands *via* a number of backbone hydrogen bonds, in a similar manner to that seen for the IFNAR1 Box2 peptide (44). This further reinforces the hypothesis that Box2 interactions are primarily driven by main chain Box2–βG1 sheet formation and burial of aliphatic sidechains into the hydrophobic core of the SH2 domain (44).

## Insights into the Basis of JAK–Receptor Specificity

**Figure 6:**
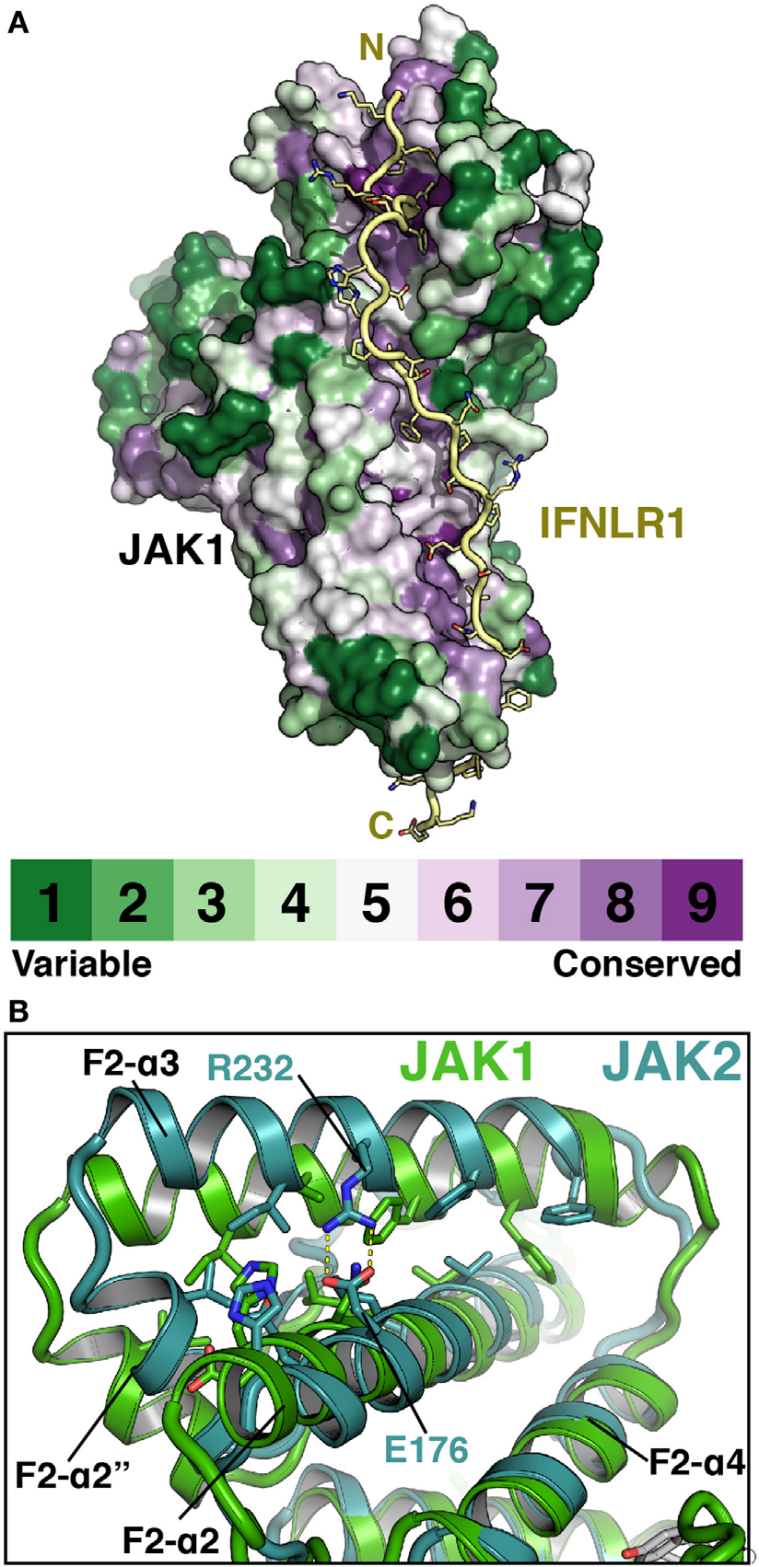
**Janus kinases (JAKs) use conserved surfaces to mediate receptor interactions**. **(A)** CONSURF analysis (55) of a ClustalO alignment of 245 JAK homologs projected onto the surface of JAK1 (PDB ID 5L04). Less conserved residues are colored in green, highly conserved residues are colored purple. IFNLR1 (yellow) shown to illustrate highly conserved residues at receptor-binding surface. **(B)** Superposition of JAK1 (green, PDB ID 5IXD) and JAK2 (cyan, PDB ID 4Z32) with a root-mean-square deviation of 1.17 Å over 1,119 atoms to illustrate shift in position of the F2-α3 and F2-α2″ helices in JAK2.

The elucidation of FERM–SH2 structures from three of the four JAK family members enables the comparison of receptor-binding surfaces of each JAK (Figure 6A). Sequence analysis of the receptor-binding interfaces on the FERM F2 and SH2 domains indicates a significant level of conservation among JAK family members, suggesting that all JAKs utilize the two common interfaces described here to interact with their receptors (Figure 6A) (44, 53, 55). While we are far from a complete understanding of key binding determinants of all JAK–receptor interactions, there are several differences between the JAK FERM F2 subdomains that may create incompatibility with certain Box1 receptor sequences. The most obvious example of this can be seen in comparison of the positioning of the F2-α3 helix between JAK1, TYK2, and JAK2. The F2-α3 helix conformation is conserved between all JAK1 and TYK2 structures available thus far, while the JAK2 F2-α3 deviates significantly (Figure 6B). A rotation of the JAK2 F2-α3 results in a shift in the N-terminus of the helix, allowing the formation of a salt bridge between Arg232 and Glu176 and an inward translation of the F2-α2″ helix. These conformational changes close off the hydrophobic pocket that, in JAK1, occupies W257 of IFNLR1. In addition to this change, differences in the linker and F2-α2 helix length observed between JAK1, TYK2, and JAK2 (Figure 1) along with sidechain variations within the peptide binding F2-α2/α3 interface suggest that the Box1 conformation seen for IFNLR1 and IL10RA bound to JAK1 may be specific to JAK1–class II receptor interactions. In other words, the class II PxxLxF sequence may be a *bona fide* JAK1-specific binding motif (Figure 4). Importantly, a different, conserved Box1 PxP motif has been found to be critical for JAK binding and functional signaling in various receptors, including gp130 (15), GHR (56), G-CSFR (57), and EPOR (28) and can seemingly interact with both JAK1 and JAK2 (Figure 4) (58). It is possible that the PxP motif (along with surrounding residues) has evolved to be a more degenerate JAK binding motif, which could explain the ability of some receptors to seemingly interact with multiple JAKs (58). Further structures of JAK1, TYK2, and/or JAK2 bound to Box1 receptor fragments will be required to answer the question of specificity versus degeneracy in JAK–Box1 interactions. Regardless, comparison of these structures suggests that the F2 subdomain is a dynamic interaction domain that can bind multiple receptor peptides, each potentially adopting a distinct conformation.

With regards to the SH2 domain, the two structures that define our understanding of SH2–Box2 interactions (TYK2–IFNAR1 and JAK1–IFNLR1) show a remarkable level of conservation in their interaction mechanisms given the low sequence homology between receptors (Figure 4). Both receptors utilize glutamate residues to interact with the vestigial pTyr binding pocket and aliphatic Box2 motifs to cap a β-hairpin in the SH2 domain. Importantly, in the apo structure of JAK2, the Box2 binding groove is open and appears to be competent for Box2 binding (Figure 5C, right panel). Therefore, with the examples in hand, it seems likely that the Box2 interaction mechanism is conserved across all JAK–receptor interactions. What is still unknown is how much specificity the Box2 brings to a JAK–receptor interaction, given the degenerate nature of Box2 interactions *via* main chain hydrogen bonding and aliphatic contacts.

## Conclusion and Perspectives

In recent years, significant progress has been made in our understanding of the structure of JAK kinases. In addition to structures of the FERM–SH2 modules and the mechanism of interaction with the receptors described in this review, structural models for the pseudokinase–kinase modules from TYK2 and JAK2 have also been revealed, providing an explanation for the mechanism of negative regulation by the pseudokinase as well as mutations linked to myeloproliferative diseases present in the psKD (35, 36). Additionally, the structure of the JAK2 kinase domain bound to its negative regulator SOCS3 has provided details on the regulation of the JAK–STAT pathway (59). Yet, despite these advances, many outstanding questions remain with regards to JAK FERM–SH2 domains and their interacting receptors. One major question is whether the FERM–SH2 has a role in activation of the kinase domain upon cytokine binding. For example, the dimerization of FERM domains from FAK has been shown to be essential to activation (60). FAK, like the JAKs, is a multi-domain tyrosine kinase containing a FERM and a kinase domain. The FAK FERM domain interacts directly with the kinase domain to mediate inhibition (61), and proximity-induced auto-phosphorylation and activation of FAK is dependent on its dimerization (60). Given that dimerization is a prerequisite to JAK activation, a role for the FERM–SH2 module in kinase activation is an intriguing possibility.

A second question is what role the enormous variation in cytokine receptor intracellular sequences plays in JAK activation (Figure 4). Even the earliest papers noted the lack of obvious consensus sequences among the intracellular domains of cytokine receptors (15). With several JAK–receptor structures now known we understand more about how receptors interact with JAKs, but it is still unclear why there is so much variation in JAK-interacting sequences. What does seem clear is that this variation is important and likely underlies important differences in JAK–receptor affinity and conformation that affects kinase activation and subsequent downstream signaling. For example, tryptophan residues within the “interbox” region (between Box1 and Box2) have been shown to disrupt JAK1 interaction and dominantly disrupt kinase signaling (26, 62–64). Yet, these Trp residues, while conserved within gp130 and EPOR isoforms, are not in the same position in relation to one another or conserved to other cytokine receptors (Figure 4). Mutations upstream of the Box1 sequence in gp130 and EPOR have also been identified that disrupt JAK signaling, but not receptor binding (65, 66). This result suggests a role for these membrane proximal sequences in facilitating proper JAK positioning to enable kinase activation. Yet, these obviously important residues are also not well conserved among receptors (Figure 4). While many questions remain unanswered, each set of unique receptor sequences undoubtedly encode a finely tuned level of kinase activity that is turned on in response to ligand binding, allowing JAK pathways to work effectively without inducing deleterious effects like cancer and autoimmunity.

The JAKs are not unique in their utilization of a FERM domain to mediate partner interactions. The FERM domain is a remarkably prolific protein interaction module, having evolved at least four separate interaction sites for both proteins and lipids. The FERM F3 subdomain, in particular, utilizes multiple surfaces to interact with a wide variety of binding partners. The ERM family members radixin and merlin along with myosin X use a shallow groove between F3-β5 and F3-α1 to interact with their partner molecules (67–70), while radixin has an additional peptide binding site formed by a pocket between F3-β4 and F3-β7 as well as a lipid interaction site between the F2 and F3 subdomains (71, 72). Additionally, myosin VIIa has been shown to use a composite interface at the junction of F1, F2, and F3 to bind to the scaffold protein SANS (73). The F2 subdomain is also involved in a number of FERM–peptide interactions. Most notably, the tumor suppressor protein merlin binds to its interaction partners LATS1/2 *via* its F2 domain (74). LATS1/2 bind to the same F2-α2, α3, and α4 groove that the IFNLR1 and IL10RA Box1 motifs were found to bind, although with opposite directionality. Merlin and moesin also contain an inhibitory CTD that binds to the FERM using a combination of the F2 and F3-β4/β7 sites (49, 74). While the evolutionary history of JAK FERM–receptor interactions will likely remain a mystery, it is tempting to speculate that an ancestral SH2-containing JAK recombined with a FERM domain that eventually facilitated conversion to a constitutive JAK–receptor interaction. Regardless, the conformational similarities and differences highlighted here reinforce the impressive plasticity and modularity of FERM and SH2 domains, both in the JAKs as well as other vital membrane-associated signaling molecules.

## Author Contributions

RF and PL wrote and edited the manuscript.

## Conflict of Interest Statement

The authors are full-time employees of Genentech, Inc.

## References

[1] Leonard WJ O’Shea JJ. Jaks and STATs: biological implications. Annu Rev Immunol (1998) 16:293–322. 10.1146/annurev.immunol.16.1.293 9597132

[2] Schwartz DM Bonelli M Gadina M O’Shea JJ. Type I/II cytokines, JAKs, and new strategies for treating autoimmune diseases. Nat Rev Rheumatol (2016) 12:25–36. 10.1038/nrrheum.2015.167 26633291 PMC4688091

[3] Brooks AJ Dai W O’Mara ML Abankwa D Chhabra Y Pelekanos RA Mechanism of activation of protein kinase JAK2 by the growth hormone receptor. Science (2014) 344:1249783. 10.1126/science.1249783 24833397

[4] Moraga I Wernig G Wilmes S Gryshkova V Richter CP Hong WJ Tuning cytokine receptor signaling by re-orienting dimer geometry with surrogate ligands. Cell (2015) 160:1196–208. 10.1016/j.cell.2015.02.011 25728669 PMC4766813

[5] Ghoreschi K Laurence A O’Shea JJ. Janus kinases in immune cell signaling. Immunol Rev (2009) 228:273–87. 10.1111/j.1600-065X.2008.00754.x 19290934 PMC2782696

[6] Haan C Kreis S Margue C Behrmann I Jaks and cytokine receptors – an intimate relationship. Biochem Pharmacol (2006) 72:1538–46. 10.1016/j.bcp.2006.04.013 16750817

[7] Quintás-Cardama A Kantarjian H Cortes J Verstovsek S. Janus kinase inhibitors for the treatment of myeloproliferative neoplasias and beyond. Nat Rev Drug Discov (2011) 10:127–40. 10.1038/nrd3264 21283107

[8] de Vos AM Ultsch M Kossiakoff AA. Human growth hormone and extracellular domain of its receptor: crystal structure of the complex. Science (1992) 255:306–12. 10.1126/science.1549776 1549776

[9] Bazan JF. Structural design and molecular evolution of a cytokine receptor superfamily. Proc Natl Acad Sci U S A (1990) 87:6934–8. 10.1073/pnas.87.18.6934 2169613 PMC54656

[10] Wang X Lupardus P Laporte SL Garcia KC. Structural biology of shared cytokine receptors. Annu Rev Immunol (2009) 27:29–60. 10.1146/annurev.immunol.24.021605.090616 18817510 PMC3981547

[11] Watowich SS Hilton DJ Lodish HF. Activation and inhibition of erythropoietin receptor function: role of receptor dimerization. Mol Cell Biol (1994) 14:3535–49. 10.1128/MCB.14.6.3535 8196600 PMC358721

[12] Renauld J-C. Class II cytokine receptors and their ligands: key antiviral and inflammatory modulators. Nat Rev Immunol (2003) 3:667–76. 10.1038/nri1153 12974481

[13] Skiniotis G Lupardus PJ Martick M Walz T Garcia KC. Structural organization of a full-length gp130/LIF-R cytokine receptor transmembrane complex. Mol Cell (2008) 31:737–48. 10.1016/j.molcel.2008.08.011 18775332 PMC2607196

[14] Fukunaga R Ishizaka-Ikeda E Pan CX Seto Y Nagata S. Functional domains of the granulocyte colony-stimulating factor receptor. EMBO J (1991) 10:2855–65. 1717255 10.1002/j.1460-2075.1991.tb07835.x PMC452996

[15] Murakami M Narazaki M Hibi M Yawata H Yasukawa K Hamaguchi M Critical cytoplasmic region of the interleukin 6 signal transducer gp130 is conserved in the cytokine receptor family. Proc Natl Acad Sci U S A (1991) 88:11349–53. 10.1073/pnas.88.24.11349 1662392 PMC53132

[16] O’Neal KD Yu-Lee LY. The proline-rich motif (PRM): a novel feature of the cytokine/hematopoietin receptor superfamily. Lymphokine Cytokine Res (1993) 12:309–12. 8260540

[17] Krolewski JJ Lee R Eddy R Shows TB Dalla-Favera R. Identification and chromosomal mapping of new human tyrosine kinase genes. Oncogene (1990) 5:277–82. 2156206

[18] Takahashi T Shirasawa T. Molecular cloning of rat JAK3, a novel member of the JAK family of protein tyrosine kinases. FEBS Lett (1994) 342:124–8. 10.1016/0014-5793(94)80485-0 8143863

[19] Harpur AG Andres AC Ziemiecki A Aston RR Wilks AF. JAK2, a third member of the JAK family of protein tyrosine kinases. Oncogene (1992) 7:1347–53. 1620548

[20] Wilks AF Harpur AG Kurban RR Ralph SJ Zürcher G Ziemiecki A. Two novel protein-tyrosine kinases, each with a second phosphotransferase-related catalytic domain, define a new class of protein kinase. Mol Cell Biol (1991) 11:2057–65. 10.1128/MCB.11.4.2057 1848670 PMC359893

[21] Müller M Briscoe J Laxton C Guschin D Ziemiecki A Silvennoinen O The protein tyrosine kinase JAK1 complements defects in interferon-alpha/beta and -gamma signal transduction. Nature (1993) 366:129–35. 10.1038/366129a0 8232552

[22] Velazquez L Fellous M Stark GR Pellegrini S. A protein tyrosine kinase in the interferon alpha/beta signaling pathway. Cell (1992) 70:313–22. 10.1016/0092-8674(92)90105-L 1386289

[23] Argetsinger LS Campbell GS Yang X Witthuhn BA Silvennoinen O Ihle JN Identification of JAK2 as a growth hormone receptor-associated tyrosine kinase. Cell (1993) 74:237–44. 10.1016/0092-8674(93)90415-M 8343952

[24] Lütticken C Wegenka UM Yuan J Buschmann J Schindler C Ziemiecki A Association of transcription factor APRF and protein kinase Jak1 with the interleukin-6 signal transducer gp130. Science (1994) 263:89–92. 10.1126/science.8272872 8272872

[25] Colamonici OR Uyttendaele H Domanski P Yan H Krolewski JJ. p135tyk2, an interferon-alpha-activated tyrosine kinase, is physically associated with an interferon-alpha receptor. J Biol Chem (1994) 269:3518–22. 8106393

[26] Witthuhn BA Quelle FW Silvennoinen O Yi T Tang B Miura O JAK2 associates with the erythropoietin receptor and is tyrosine phosphorylated and activated following stimulation with erythropoietin. Cell (1993) 74:227–36. 10.1016/0092-8674(93)90414-L 8343951

[27] Miyazaki T Kawahara A Fujii H Nakagawa Y Minami Y Liu ZJ Functional activation of Jak1 and Jak3 by selective association with IL-2 receptor subunits. Science (1994) 266:1045–7. 10.1126/science.7973659 7973659

[28] Huang LJ Constantinescu SN Lodish HF. The N-terminal domain of Janus kinase 2 is required for Golgi processing and cell surface expression of erythropoietin receptor. Mol Cell (2001) 8:1327–38. 10.1016/S1097-2765(01)00401-4 11779507

[29] Frank SJ Yi W Zhao Y Goldsmith JF Gilliland G Jiang J Regions of the JAK2 tyrosine kinase required for coupling to the growth hormone receptor. J Biol Chem (1995) 270:14776–85. 10.1074/jbc.270.24.14776 7540178

[30] Zhao Y Wagner F Frank SJ Kraft AS. The amino-terminal portion of the JAK2 protein kinase is necessary for binding and phosphorylation of the granulocyte-macrophage colony-stimulating factor receptor beta c chain. J Biol Chem (1995) 270:13814–8. 10.1074/jbc.270.23.13814 7775438

[31] Velazquez L Mogensen KE Barbieri G Fellous M Uzé G Pellegrini S. Distinct domains of the protein tyrosine kinase tyk2 required for binding of interferon-alpha/beta and for signal transduction. J Biol Chem (1995) 270:3327–34. 10.1074/jbc.270.7.3327 7531704

[32] Yan H Piazza F Krishnan K Pine R Krolewski JJ. Definition of the interferon-alpha receptor-binding domain on the TYK2 kinase. J Biol Chem (1998) 273:4046–51. 10.1074/jbc.273.7.4046 9461596

[33] Richter MF Duménil G Uzé G Fellous M Pellegrini S. Specific contribution of Tyk2 JH regions to the binding and the expression of the interferon alpha/beta receptor component IFNAR1. J Biol Chem (1998) 273:24723–9. 10.1074/jbc.273.38.24723 9733772

[34] Chen M Cheng A Chen YQ Hymel A Hanson EP Kimmel L The amino terminus of JAK3 is necessary and sufficient for binding to the common gamma chain and confers the ability to transmit interleukin 2-mediated signals. Proc Natl Acad Sci U S A (1997) 94:6910–5. 10.1073/pnas.94.13.6910 9192665 PMC21258

[35] Lupardus PJ Ultsch M Wallweber H Bir Kohli P Johnson AR Eigenbrot C. Structure of the pseudokinase-kinase domains from protein kinase TYK2 reveals a mechanism for Janus kinase (JAK) autoinhibition. Proc Natl Acad Sci U S A (2014) 111:8025–30. 10.1073/pnas.1401180111 24843152 PMC4050602

[36] Shan Y Gnanasambandan K Ungureanu D Kim ET Hammarén H Yamashita K Molecular basis for pseudokinase-dependent autoinhibition of JAK2 tyrosine kinase. Nat Struct Mol Biol (2014) 21:579–84. 10.1038/nsmb.2849 24918548 PMC4508010

[37] Luo H Rose P Barber D Hanratty WP Lee S Roberts TM Mutation in the Jak kinase JH2 domain hyperactivates *Drosophila* and mammalian Jak-Stat pathways. Mol Cell Biol (1997) 17:1562–71. 10.1128/MCB.17.3.1562 9032284 PMC231882

[38] Saharinen P Takaluoma K Silvennoinen O. Regulation of the Jak2 tyrosine kinase by its pseudokinase domain. Mol Cell Biol (2000) 20:3387–95. 10.1128/MCB.20.10.3387-3395.2000 10779328 PMC85631

[39] Casanova JL Holland SM Notarangelo LD. Inborn errors of human JAKs and STATs. Immunity (2012) 36:515–28. 10.1016/j.immuni.2012.03.016 22520845 PMC3334867

[40] Cacalano NA Migone TS Bazan F Hanson EP Chen M Candotti F Autosomal SCID caused by a point mutation in the N-terminus of Jak3: mapping of the Jak3-receptor interaction domain. EMBO J (1999) 18:1549–58. 10.1093/emboj/18.6.1549 10075926 PMC1171243

[41] Schmalstieg FC Leonard WJ Noguchi M Berg M Rudloff HE Denney RM Missense mutation in exon 7 of the common gamma chain gene causes a moderate form of X-linked combined immunodeficiency. J Clin Invest (1995) 95:1169–73. 10.1172/JCI117765 7883965 PMC441454

[42] Russell SM Johnston JA Noguchi M Kawamura M Bacon CM Friedmann M Interaction of IL-2R beta and gamma c chains with Jak1 and Jak3: implications for XSCID and XCID. Science (1994) 266:1042–5. 10.1126/science.7973658 7973658

[43] Lupardus PJ Skiniotis G Rice AJ Thomas C Fischer S Walz T Structural snapshots of full-length Jak1, a transmembrane gp130/IL-6/IL-6Rα cytokine receptor complex, and the receptor-Jak1 holocomplex. Structure (2011) 19:45–55. 10.1016/j.str.2010.10.010 21220115 PMC3052743

[44] Wallweber HJA Tam C Franke Y Starovasnik MA Lupardus PJ Structural basis of recognition of interferon-α receptor by tyrosine kinase 2. Nat Struct Mol Biol (2014) 21:443–8. 10.1038/nsmb.2807 24704786 PMC4161281

[45] Ferrao R Wallweber HJA Ho H Tam C Franke Y Quinn J The structural basis for class II cytokine receptor recognition by JAK1. Structure (2016) 24:897–905. 10.1016/j.str.2016.03.023 27133025 PMC4899269

[46] Zhang D Wlodawer A Lubkowski J Crystal structure of a complex of the intracellular domain of interferon λ receptor 1 (IFNLR1) and the FERM/SH2 domains of human JAK1. J Mol Biol (2016) 428(23):4651–68. 10.1016/j.jmb.2016.10.005 27725180 PMC6288811

[47] McNally R Toms AV Eck MJ. Crystal structure of the FERM-SH2 module of human Jak2. PLoS One (2016) 11:e0156218. 10.1371/journal.pone.0156218 27227461 PMC4881981

[48] Ceccarelli DFJ Song HK Poy F Schaller MD Eck MJ. Crystal structure of the FERM domain of focal adhesion kinase. J Biol Chem (2006) 281:252–9. 10.1074/jbc.M509188200 16221668

[49] Pearson MA Reczek D Bretscher A Karplus PA. Structure of the ERM protein moesin reveals the FERM domain fold masked by an extended actin binding tail domain. Cell (2000) 101:259–70. 10.1016/S0092-8674(00)80836-3 10847681

[50] Bradshaw JM Waksman G. Molecular recognition by SH2 domains. Adv Protein Chem (2002) 61:161–210. 10.1016/S0065-3233(02)61005-8 12461824

[51] Ragimbeau J Dondi E Alcover A Eid P Uzé G Pellegrini S. The tyrosine kinase Tyk2 controls IFNAR1 cell surface expression. EMBO J (2003) 22:537–47. 10.1093/emboj/cdg038 12554654 PMC140723

[52] Radtke S Haan S Jörissen A Hermanns HM Diefenbach S Smyczek T The Jak1 SH2 domain does not fulfill a classical SH2 function in Jak/STAT signaling but plays a structural role for receptor interaction and up-regulation of receptor surface expression. J Biol Chem (2005) 280:25760–8. 10.1074/jbc.M500822200 15894543

[53] McNally R Eck MJ JAK-cytokine receptor recognition, unboxed. Nat Struct Mol Biol (2014) 21:431–3. 10.1038/nsmb.2824 24799036

[54] Haan S Margue C Engrand A Rolvering C Schmitz-Van de Leur H Heinrich PC Dual role of the Jak1 FERM and kinase domains in cytokine receptor binding and in stimulation-dependent Jak activation. J Immunol (2008) 180:998–1007. 10.4049/jimmunol.180.2.998 18178840

[55] Ashkenazy H Abadi S Martz E Chay O Mayrose I Pupko T ConSurf 2016: an improved methodology to estimate and visualize evolutionary conservation in macromolecules. Nucleic Acids Res (2016) 44:W344–50. 10.1093/nar/gkw408 27166375 PMC4987940

[56] Dinerstein H Lago F Goujon L Ferrag F Esposito N Finidori J The proline-rich region of the GH receptor is essential for JAK2 phosphorylation, activation of cell proliferation, and gene transcription. Mol Endocrinol (1995) 9:1701–7. 10.1210/mend.9.12.8614406 8614406

[57] Avalos BR Hunter MG Parker JM Ceselski SK Druker BJ Corey SJ Point mutations in the conserved box 1 region inactivate the human granulocyte colony-stimulating factor receptor for growth signal transduction and tyrosine phosphorylation of p75c-rel. Blood (1995) 85:3117–26. 7538818

[58] Stahl N Boulton TG Farruggella T Ip NY Davis S Witthuhn BA Association and activation of Jak-Tyk kinases by CNTF-LIF-OSM-IL-6 beta receptor components. Science (1994) 263:92–5. 10.1126/science.8272873 8272873

[59] Kershaw NJ Murphy JM Liau NPD Varghese LN Laktyushin A Whitlock EL SOCS3 binds specific receptor-JAK complexes to control cytokine signaling by direct kinase inhibition. Nat Struct Mol Biol (2013) 20:469–76. 10.1038/nsmb.2519 23454976 PMC3618588

[60] Brami-Cherrier K Gervasi N Arsenieva D Walkiewicz K Boutterin M-C Ortega A FAK dimerization controls its kinase-dependent functions at focal adhesions. EMBO J (2014) 33:356–70. 10.1002/embj.201386399 24480479 PMC3989642

[61] Lietha D Cai X Ceccarelli DFJ Li Y Schaller MD Eck MJ. Structural basis for the autoinhibition of focal adhesion kinase. Cell (2007) 129:1177–87. 10.1016/j.cell.2007.05.041 17574028 PMC2077847

[62] Watowich SS Liu KD Xie X Lai SY Mikami A Longmore GD Oligomerization and scaffolding functions of the erythropoietin receptor cytoplasmic tail. J Biol Chem (1999) 274:5415–21. 10.1074/jbc.274.9.5415 10026152 PMC2388248

[63] Haan C Hermanns HM Heinrich PC Behrmann I A single amino acid substitution (Trp(666)->Ala) in the interbox1/2 region of the interleukin-6 signal transducer gp130 abrogates binding of JAK1, and dominantly impairs signal transduction. Biochem J (2000) 349:261–6. 10.1042/0264-6021:3490261 10861237 PMC1221146

[64] Miura O Cleveland JL Ihle JN Inactivation of erythropoietin receptor function by point mutations in a region having homology with other cytokine receptors. Mol Cell Biol (1993) 13:1788–95. 10.1128/MCB.13.3.1788 8382775 PMC359491

[65] Haan C Heinrich PC Behrmann I. Structural requirements of the interleukin-6 signal transducer gp130 for its interaction with Janus kinase 1: the receptor is crucial for kinase activation. Biochem J (2002) 361:105–11. 10.1042/0264-6021:3610105 11742534 PMC1222284

[66] Constantinescu SN Huang LJ-S Nam H-S Lodish HF. The erythropoietin receptor cytosolic juxtamembrane domain contains an essential, precisely oriented, hydrophobic motif. Mol Cell (2001) 7:377–85. 10.1016/S1097-2765(01)00185-X 11239466

[67] Takai Y Kitano K Terawaki S-I Maesaki R Hakoshima T. Structural basis of the cytoplasmic tail of adhesion molecule CD43 and its binding to ERM proteins. J Mol Biol (2008) 381:634–44. 10.1016/j.jmb.2008.05.085 18614175

[68] Hamada K Shimizu T Yonemura S Tsukita S Tsukita S Hakoshima T. Structural basis of adhesion-molecule recognition by ERM proteins revealed by the crystal structure of the radixin-ICAM-2 complex. EMBO J (2003) 22:502–14. 10.1093/emboj/cdg039 12554651 PMC140724

[69] Wei Z Yan J Lu Q Pan L Zhang M. Cargo recognition mechanism of myosin X revealed by the structure of its tail MyTH4-FERM tandem in complex with the DCC P3 domain. Proc Natl Acad Sci U S A (2011) 108:3572–7. 10.1073/pnas.1016567108 21321230 PMC3048157

[70] Li Y Wei Z Zhang J Yang Z Zhang M. Structural basis of the binding of merlin FERM domain to the E3 ubiquitin ligase substrate adaptor DCAF1. J Biol Chem (2014) 289:14674–81. 10.1074/jbc.M114.551184 24706749 PMC4031523

[71] Terawaki S-I Maesaki R Hakoshima T. Structural basis for NHERF recognition by ERM proteins. Structure (2006) 14:777–89. 10.1016/j.str.2006.01.015 16615918

[72] Hamada K Shimizu T Matsui T Tsukita S Hakoshima T. Structural basis of the membrane-targeting and unmasking mechanisms of the radixin FERM domain. EMBO J (2000) 19:4449–62. 10.1093/emboj/19.17.4449 10970839 PMC302071

[73] Wu L Pan L Wei Z Zhang M. Structure of MyTH4-FERM domains in myosin VIIa tail bound to cargo. Science (2011) 331:757–60. 10.1126/science.1198848 21311020

[74] Li Y Zhou H Li F Chan SW Lin Z Wei Z Angiomotin binding-induced activation of merlin/NF2 in the Hippo pathway. Cell Res (2015) 25:801–17. 10.1038/cr.2015.69 26045165 PMC4493278

